# Supplementary material for: Galectin and Myc enable cochlear progenitor expansion in vitro and in vivo
Source: bioRxiv. 2026 Jun 7:2026.06.03.729765. Preprint. [Version 1] doi: 10.64898/2026.06.03.729765 (PMC13251994; doi:10.64898/2026.06.03.729765)
Supplement: 1 [file NIHPP2026.06.03.729765v1-supplement-1.pdf]

bioRxiv preprint doi: https://doi.org/10.64898/2026.06.03.729765; this version posted June 7, 2026. The copyright holder for this preprint (which was not certified by peer review) is the author/funder, who has granted bioRxiv a license to display the preprint in perpetuity. It is made available under a CC-BY 4.0 International license.

Table S1: Marker genes for clusters 1-7 in GER-derived organoids.

Table S2: Ingenuity pathway analysis results – top 10 pathways.

Table S3: Differentially expressed genes at the onset of proliferation.

Table S4: Marker genes for CellTrails clusters in GER-derived organoids.

Table S5: Differentially expressed genes at the onset of proliferation for CellTrails analysis.

Table S6: Ingenuity pathway analysis results for CellTrails analysis – top 10 pathways.

Table S7: Differentially expressed genes in organ of Corti cells.

Table S8: Differentially expressed genes in *in vivo* damage model (Lgr5-DTR) and control.
